# Unusual Non-Occupational Exposure to Metals

**DOI:** 10.1155/S1565363303000049

**Published:** 2003

**Authors:** Renate Wrbitzky

**Affiliations:** 1 Department of Occupational Medicine of the Medical School of Hanover Hanover 30623 Germany

## Abstract

Exposure to metals at workplaces is well known and in many cases occupational studies led to an adoption of limit values. For airborne concentrations of substances as metals refer to the “Maximaleo Arbeitsplatz-Konzentration” (MAK) in Germany or the “Threshold Limit Value” (TLV) in USA. Biological monitoring consists of an assessment of overall exposure to chemicals at the workplace and in the environment. The “Biologischer Arbeitsstoff Toleranzwert” (BAT) in Germany and the “Biological Exposure Index” in the USA serve as reference values. Besides these occupational limit values, reference values exist in Germany for the background exposure of the non occupationally exposed general population. In some cases the reference values are exceeded without any occupational exposure. Several cases of unusual environmental exposure to cobalt, mercury and manganese are reported. In such cases, it is often difficult to evaluate the measured concentration. In Germany, therefore, the “Human-Biomonitoring-Werte” (HBMValues) have been adopted in order to evaluate such high background exposures. The HBM-concept is presented. Environmental exposure to metals is usual within some limits. Reference values are helpful for an assessment. Unusual exposure occurs and the physician should be alert to symptoms of poisoning.


### Unusual Non-Occupational Exposure to Metals

Prof. Dr. med. Renate Wrbitzky

*Department of Occupational Medicine of the Medical School of Hanover*
*30623 Hanover, Germany*
*Fax: ++49 511 5329330*
*e-mail: wrbitzky.renate@mh-hannover.de*

(Received: October 10, 2002; Accepted: December 9, 2002)

### ABSTRACT

Exposure to metals at workplaces is well known and in many cases occupational studies led to an adoption of limit values. For airborne concentrations of substances as metals refer to the "Maximale-Arbeitsplatz-Konzentration" (MAK) in Germany or the "Threshold Limit Value" (TLV) in USA. Biological monitoring consists of an assessment of overall exposure to chemicals at the workplace and in the environment. The "Biologischer Arbeitsstoff Toleranzwert" (BAT) in Germany and the "Biological Exposure Index" in the USA serve as reference values. Besides these occupational limit values, reference values exist in Germany for the background exposure of the non occupationally exposed general population. In some cases the reference values are exceeded without any occupational exposure. Several cases of unusual environmental exposure to cobalt, mercury and manganese are reported. In such cases, it is often difficult to evaluate the measured concentration. In Germany, therefore, the "Human-Biomonitoring-Werte" (HBM-Values) have been adopted in order to evaluate such high background exposures. The HBM-concept is presented. Environmental exposure to metals is usual within some limits. Reference values are helpful for an assessment. Unusual exposure occurs and the physician should be alert to symptoms of poisoning.

### INTRODUCTION

Exposure to metals at workplaces is well known and in many cases occupational studies have led to an adoption of limit values. For airborne concentrations of substances as metals refer to the "Maximale-Arbeitsplatz-Konzentration" (MAK) in Germany or the "Threshold Limit Value" (TLV) in the USA /1,2/. Biological monitoring consists of an assessment of overall exposure to chemicals at the workplace and in the environment. The "Biologischer Arbeitsstoff Toleranzwert" (BAT) in Germany /3/ and the "Biological Exposure Index" in USA serve as reference values.

Besides these occupational limit values, reference values exist in Germany for the background exposure of the non occupationally exposed general population /4,5/. These values are officially accepted and confirmed. However, reference values are established statistically. The reference point is the ninety fifth percentile. Ninety-five percent of the values in a representative group of the general population have concentrations below, while 5% exceed the reference value. These values only describe statistically the body's chemical load, they do not distinguish between hazardous or non-hazardous. Reference values can vary between regions and countries. They can change: The values for polychlorinated biphenyls or dioxins, for instance, have decreased in recent years /6/.

Table 1 summarizes the reference values for some metals, describing the background exposure of the general population in Germany. Usually the concentrations are measured in blood or urine /4,5/.

In some cases the reference values are exceeded without any occupational exposure. In the following several cases of unusual environmental exposure to lead, cobalt, manganese, mercury and amalgam are reported.

**Table 1**

Reference values for metals describing the background exposure of the general population in Germany /4,5/

| PARAMETER | REFERENCE-VALUE | MATRIX |
|---|---|---|
| aluminium | < 2000 µg/ g Cr. | urine |
| lead | 60 – 120 µg/l | blood |
| cobalt | < 2,0 µg/g Cr. | urine |
| copper | 50 µg/g Cr. | urine |
| manganese | 10-12 µg/l | blood |
| mercury | < 10 µg/l | urine |

**CASE REPORTS:**

### Lead

In 1998 a female patient complained of dizziness, headache and abdominal pain. Environmental investigation showed that she had bought in the previous year some items of kitchen pottery, which she used almost daily, during her holidays in the Mediterranean area. It is well known that these articles of pottery may have glazes containing lead. Biomonitoring revealed a lead exposure about 300 µg/l in blood. This concentration is much higher than the background level and reaches the BAT value at workplaces /2/. The blood lead levels decreased after the pottery had been disposed of.

In another case of environmental lead exposure a male patient suffered a shotgun injury. The x-ray shows the shotgun injury with deposition of several pellets containing lead (Fig. 1). The investigations of blood lead concentrations showed a significant increase of lead within the following 6 days. The maximum was 720 µg/l blood. The reference value of lead for environmental exposure is between 60 and 120 µg/l /4/. Another marker for lead exposition is the Delta-Aminolaevulin Acid in urine, which corresponded in this case to the increasing and decreasing lead levels and showed the dynamics of lead exposure.

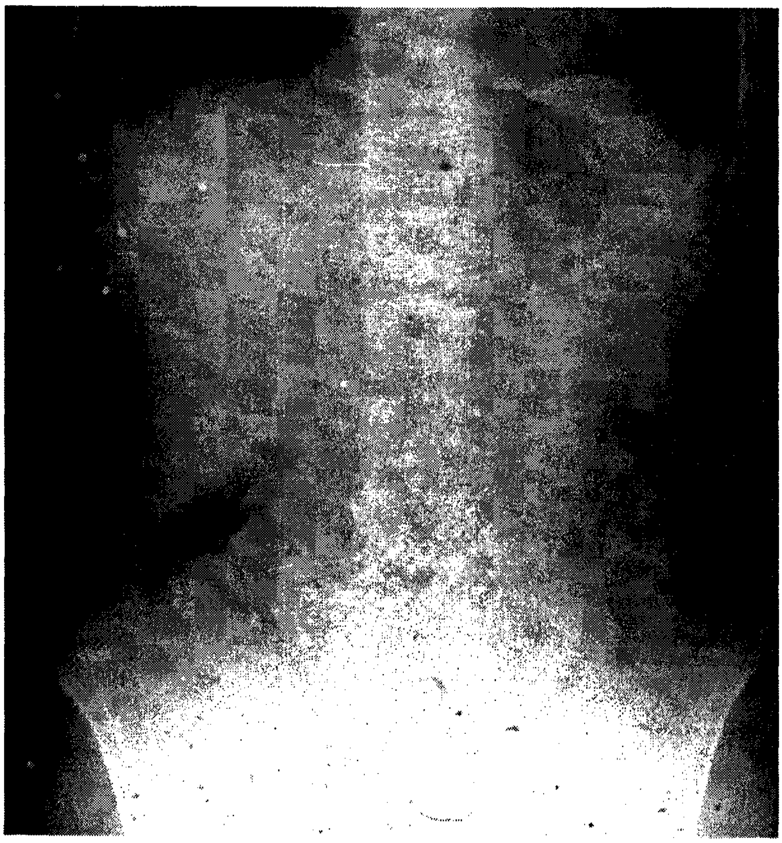

**Fig. 1:** Shotgun injury with deposition of several pellets (right thorax and hip) containing lead (Out-Patient-Clinic of Occupational-, Social- and Environmental Medicine, University of Erlangen-Nuremberg)

### Cobalt

Cobalt exposure is usual at some workplaces with heavy metal processes. Cobalt is known to produce cough, dyspnoea, wheezing, asthma or interstitial fibrosis, known as "hard metal disease". In this case, following hip hemiarthroplasty, a patient suffered from a cardiomyopathy that was described as the effect of cobalt. As the arthroplasty contained cobalt and other metals a metal analysis was carried out, which revealed high cobalt and chromium levels. The level of cobalt in blood was 230.0 µg/l – much higher than the actual German BAT value of 5 µg/l /2/. The blood value of chromium was 17.0 µg/l – within the actual EKA value of 35.0 µg/l /2/. Evidently due to an infection, which was clinically confirmed and radiologically assessed, the arthroplasty dissolved and caused the high internal exposure. Further investigation showed no environmental or occupational exposure. In conclusion, if there is an infection of an arthroplasty the physician should consider a potential release of metals.

### Manganese

A 54-year-old woman complained of non-specific symptoms such as headache, dizziness, loss of concentration. Organic diseases were not found. As she was convinced that she had been exposed to heavy metals in her drinking water, some relevant analyses were done: lead, copper and manganese in blood. The concentrations of lead and copper were within the limits of the background level, but the manganese, at 18 µg/l blood, was slightly above the reference value of the general population, which is about 12 µg/l in Germany. This value nearly reaches the German BAT value for manganese, which is determined as 20 µg/l /2/. We did not find any explanation for this unusual exposure. Drinking water was controlled and without high concentrations of manganese.

Nutritional habits were normal. There was no high uptake of seaweed or algae, which are known to show high concentrations of manganese. A repeat blood concentration half a year later was, at 16.8 µg/l, nearly unchanged.

Even if the symptoms of the patient differ from the symptoms that are reported from higher manganese exposure (e.g., increased tremor and decreased short-term memory) it is problematic that the German BAT value for working places is nearly reached. This value was determined due to the above mentioned neurological effects of manganese which were found in some studies /2, 7/.

### Metallic Mercury

Winker *et al.* of the Division of Occupational Medicine in Vienna, Austria, reported an extraordinary case of poisoning with metallic mercury /8/: A previously drug-addicted patient intravenously injected himself with about 8 g (0.6 ml) of metallic mercury in an attempt at suicide and took about 100 ml orally. He was working in an incinerating plant and acquired the bottle at his workplace. Within a few days he developed typical symptoms of acute mercury intoxication, such as gastroenteritis, colitis, stomatitis and metallic taste in the mouth. About 2 weeks later he showed a strong tremor in the hands, exhaustion and insomnia. Six weeks later he was examined and complained of lack of concentration, speech disorder and tremor.

Mercury concentration in blood was 680 µg/l as compared to the reference value of about 20 µg/l. Mercury concentration in urine was 140 µg/l, corresponding to a reference value of 10 µg/l. The clinical investigations showed no abnormal results (velocity of nerve conduction, electromyography, electroencephalogram). Chelation therapy was done and mercury levels decreased to 478 µg/l.

As expected, the x-ray examination showed extensive amounts of metallic mercury droplets within the original lesions. Three years after the suicide attempt an extirpation of residual mercury was carried out. About 2 g of the injected mercury could be removed, and the mercury blood levels then decreased to about 250 µg/l. Further observations showed no symptoms of chronic mercury intoxication. Metallic mercury does not have the same toxic effects as organic or inorganic mercury due to another bioavailability. Acute lethal consequences are not expected in such cases. Long term experience however is lacking in most cases /9/.

### Amalgam

Some patients argue that the release of mercury from amalgam fillings is causally connected with their complaints and diseases. A recently published study determined the internal exposure to mercury in 2 groups differing in their attitude towards possible health hazards by mercury /10/. Forty females suffered from serious health damage due to amalgam fillings, 43 female control subjects did not claim any association.

Median mercury blood levels were 2.35 (0.25-13.40) µg/l for the "amalgam sensitive group" and 2.40 (0.25-14.70) µg/l for "amalgam non-sensitive group".

Mercury levels in blood and urine were within the range of background levels in the general population including persons with amalgam fillings. No statistical difference was found between both groups with regard to internal mercury exposure. Nevertheless amalgam sensitive patients suffer from various diseases and are convinced that amalgam fillings have caused them.

### Concept of the "Human-Biomonitoring-Values"

In such cases, even if the exposure is within the range of the background exposure, it is often difficult to evaluate the measured concentration. In Germany, therefore, the "Human-Biomonitoring-Werte" (HBM-Values) have been adopted in order to evaluate actual exposure and especially high background exposures.

The Human Biomonitoring Commission has defined HBM-I values and HBM-II values for different pollutants and metals. The HBM values represent health related exposure limits.

### *HBM-I Value*

According to the Commission the HBM-I value is a value below which a risk of adverse effects in the general population is not to be expected according to current knowledge.

### *HBM-II Value*

Adverse effects cannot be excluded with sufficient certainty for concentrations in the range between HBM-I and HBM-II value. A general health risk is not expected but follow-up is suggested.

If a result exceeds the HBM-II value, there is a possibility of an increased risk of adverse health effects with the necessity to reduce exposure. Table 2 shows the HBM-values for lead, cadmium and mercury.

For the individual risk assessment it is possible to use the German occupational limit values. These occupational limit values are defined as exposure limits which distinguish between non hazardous and hazardous exposure.

**Table 2**

Human Biomonitoring Values for selected substances /11,12,13/

| | HBM I | HBM II |
|---|---|---|
| lead (blood) < 12 y.; f < 45 y.<br>> 45 y. | 100 µg/l<br>150 µg/l | 150 µg/l<br>250 µg/l |
| cadmium (urine) < 25 y.<br>> 25 y. | 1 µg/g Cr.<br>2 µg/g Cr. | 3 µg/g Cr.<br>5 µg/g Cr. |
| mercury (urine)<br>(blood) | 5 µg/g Cr.<br>5 µg/l | 20 µg/g Cr.<br>15 µg/l |

f = females; y = year; Cr. = creatinine

### CONCLUSIONS

Unusual exposure to metals occurs and the physician should be alert to symptoms of poisoning. Reference values are helpful for an assessment of environmental exposure to metals. However, these values only describe statistically the body's chemical load, they do not distinguish between hazardous or non-hazardous. Therefore it is useful to evaluate HBM values, which distinguish between no risk and increased risk of adverse health effects. As the number of evaluated HBM values is small, the occupational limit values can be used for comparison of exposure. The occupational limit values are scientifically evaluated and defined as limits below which no health effects are expected. But it must be considered, that they relate to healthy workers and not to the general population including children or sick and elderly people. For a risk assessment of non occupational exposure to metals it might be helpful to consider all existing exposure values.

### ACKNOWLEDGEMENT:

I would like to thank Prof. Dr. Dr. h.c. G. Lehnert (em. Dir. of the Out-Patient Clinic for Occupational-, Social- and Environmental Medicine of the University Erlangen-Nuremberg) for use of the X-ray.

### REFERENCES

1. American Conference of Governmental Industrial Hygienists (ACGIH). *Threshold Limit Values for Chemical Substances and Physical Agents and Biological Exposure Indices 1999/2000,* Cincinnati (2001).
2. H Greim (Hrsg.). *MAK- und BAT-Werte-Liste 2002.* Senatskommission zur Prüfung gesundheitsschädlicher Arbeitsstoffe. Mitteilung **38**. Wiley-VCH Verlagsgesellschaft, Weinheim.
3. H Greim and G Lehnert (Hrsg.): *Biologische Arbeitsstoff-Toleranzwerte (BAT-Werte) und Expositionsäquivalente für krebserzeugende Arbeitsstoffe (EKA).* Arbeitsgruppe Aufstellung von Grenzwerten in biologischem Material der Senatskommission zur Prüfung gesundheitsschädlicher Arbeitsstoffe der Deutschen Forschungsgemeinschaft. Wiley-VCH Verlagsgesellschaft, Weinheim.
4. K Becker, S Kaus, C Krause, P Lepom, C Schulz, M Seiwert and B Seifert. German Environmental Survey 1998: environmental pollutants in blood of the German population, *Int. J. Hyg. Environ. Health,* **205**, 297-308 (2002).
5. R Wrbitzky, T Göen , S Letzel, F Frank and J Angerer. Internal exposure of waste incineration workers to organic and inorganic substances. *Int Arch Occup Environ Health*, **68**, 13-21 (1995).
6. R Wrbitzky, B Flatau, B Beyer, H Thoma, M Hennig, A Weber, J Angerer and G Lehnert. Internal exposure to polychlorinated dibenzo-p-dioxins and polychlorinated dibenzofurans (PCDDs/PCDFs) of Bavarian chimney sweeps. *Arch. Environ. Contam. Toxicol.*, **40**, 136-140 (2001).

7. M Bader, MC Dietz, A Ihrig and G Triebig: Biomonitoring of manganese in blood, urine and axillary hair following low-dose exposure during the manufacture of dry cell batteries. *Int-Arch Occup Environ Health*, **72**(8), 521-7 (1999).
8. R Winker, AW Schaffer, C Konnaris, A Barth, P Giovanoli, HW Rüdiger and C Wolf: Health consequences of an intravenous injection of metallic mercury. *Int Arch Occup Environ Health*, **75**, 587-590 (2002).
9. RJ Giombetti, DH Rosen, AR Kuczmierczyk and DO Marsh: Repeated suicide attempts by the intravenous injection of elemental mercury. *Int J Psychiatry Med*, **18**, 153-167 (1988).
10. H Zimmer, H Ludwig, M Bader, J Bailer, P Eickholz, HJ Staehle and G Triebig: Determination of mercury in blood, urine and saliva for the biological monitoring of an exposure from amalgam fillings in a group with self-reported adverse health effects. *Int.J. Hyg. Environ. Health*, **205**, 205-211 (2002).
11. HBM/UBA (Kommission "Human-Biomonitoring des Umweltbundesamtes): Konzept der Referenz- und Human-Biomonitoring-Werte (HBM) in der Umweltmedizin. *Bundesgesundhbl*; **6**, 221-24 (1996).
12. HBM-UBA (Kommission Human-Biomonitoring des Umweltbundesamtes): Stoffmonographie Quecksilber – Referenz- und Human-Biomonitoring-Werte (HBM). *Bundesgesundhbl.* **42** (6), 522-532 (1999).
13. HBM-UBA (Kommission Human-Biomonitoring des Umweltbundesamtes). Human-Biomonitoring: Definition, Möglichkeiten und Voraussetzungen. *Bundesgesundhbl* **6**, 213-215 (1996).

